# Arthroscopic arthrolysis of posttraumatic and non-traumatic elbow stiffness offers comparable clinical outcomes

**DOI:** 10.1186/s12891-019-2666-1

**Published:** 2019-06-15

**Authors:** Saroj Rai, Qimin Zhang, Nira Tamang, Shengyang Jin, Hong Wang, Chunqing Meng

**Affiliations:** 1 Department of Orthopaedics, Union Hospital, Tongji Medical College, Huazhong University of Science and Technology, Wuhan, 430022 China; 2 Department of Orthopaedics, National Trauma Center, National Academy of Medical Sciences, Kathmandu, Nepal; 3 School of Nursing, Tongji Medical College, Huazhong University of Science and Technology, Wuhan, 430030 China; 4 Norvic International Hospital, Kathmandu, Nepal

**Keywords:** Arthroscopic, Arthrolysis, Elbow, Stiffness, Posttraumatic, Non-traumatic

## Abstract

**Background:**

Primary purpose of this study is to compare the clinical outcomes of patients undergoing arthroscopic arthrolysis in posttraumatic and non-traumatic elbow stiffness. Secondary aims are to compare the level of satisfaction and complications.

**Methods:**

We retrospectively evaluated the patients undergoing arthroscopic elbow arthrolysis between January 2008 and September 2015 and have completed a minimum 2-year follow-up. Total of 141 patients (male = 90; female = 51) with 143 elbows (posttraumatic, *n* = 75; non-traumatic, *n* = 68) with an average age of 33 years were available for final evaluation. The average follow-up period was 44 months. We used the Mayo Elbow Performance Index (MEPI) score, range of motion (ROM), Visual Analogue Scale (VAS) to measure clinical outcomes. The level of satisfaction was measured by a self-constructed questionnaire.

**Results:**

All parameters were significantly improved postoperatively (*P* < 0.01). However, statistically significant differences were not present in the rate of postoperative improvement of elbow ROM (*P* = 0.08) and MEPI (*P* = 0.21) in both groups. According to MEPI, 72(96%) elbows in posttraumatic and 60(88%) elbows in non-traumatic group were rated as good to excellent. No statistically significant differences were observed in the level of satisfaction (*P* = 0.76) and rate of complications (*P* = 0.91).

**Conclusions:**

Arthroscopic arthrolysis is an effective tool and a good option for the treatment of patients with posttraumatic and non-traumatic elbow stiffness. The rate of elbow ROM and MEPI score improvements were significant and comparable postoperatively with a high level of patient’s satisfaction. However, postoperative rehabilitation is equally essential to maintain intraoperative elbow ROM, to attain optimal outcome and to prevent complications.

## Background

According to Morrey and colleague [1], an elbow joint should have at least 30° - 130° of extension-flexion, and 50° - 50° pronation-supination movement to function normally. Handling some modern equipment such as a computer mouse and typing on a keyboard requires more pronation, and using a mobile phone even requires elbow flexion up to 149° [2]. The range of motion (ROM) of less than 100° and/or more than 30°of flexion contracture is regarded as elbow stiffness and is a fairly common condition that significantly affects the patient’s quality of life (QoL) [1]. Fifty degrees of loss of elbow motion results in 80% loss of function that requires for activities of daily living (ADL) [1]. The aetiology of elbow stiffness is considered to be multifactorial and can broadly be divided into traumatic and non-traumatic elbow stiffness [3–5].

The ROM and functional improvement are the primary goals of elbow arthrolysis. The general indication of arthrolysis is failed non-operative treatment having less than 100° of elbow ROM or less than 130° flexion contracture and/or more than 30° extension contracture [6, 7]. However, indications can appropriately be relaxed on the basis of the patient’s occupation and need [6]. Both open and arthroscopic arthrolysis can reproduce a significant postoperative clinical improvement [3–9]. Open arthrolysis is effective even in severe stiffness; however, it is associated with relatively higher complications [10]. Recently, arthroscopic arthrolysis has been introduced as a minimally invasive procedure for the treatment of elbow stiffness and is gaining popularity among arthroscopic surgeons [5, 7, 8, 10–13]. It allows very precise and selective removal of scar tissues or osteophytes or any intra-articular pathology with minimal trauma and also reported to have lower complications [5, 8, 10, 14]. That adds to rapid recovery and earlier rehabilitation [4, 7]. However, most of the previous reports focused mainly on posttraumatic stiffness with minimal ROM restrictions [7, 12, 14–18]. The reports on non-traumatic stiffness or comparison between the posttraumatic and non-traumatic stiffness are still scarce [3–5, 8, 11].

The primary aim of this study is to compare the clinical outcomes in terms of ROM and function in patients undergoing arthroscopic arthrolysis for posttraumatic and non-traumatic stiffness with a minimum follow-up of 2-year. The secondary aims are to compare the level of satisfaction and complications. We hypothesized that there would be no difference in the clinical outcomes between the two groups.

## Methods

We conducted a retrospective review of patients who underwent arthroscopic arthrolysis of elbow stiffness between January 2008 and September 2015. Patients are included in this study if elbow stiffness (ROM< 100°) with failed non-operative management with physiotherapy for at least 6 months that significantly affected the patient’s ADL. The exclusion criteria were as follows: 1). Stiffness secondary to neuromuscular diseases such as cerebral palsy, muscle spasticity, and chronic regional pain syndrome, 2). Gross anatomical deformity and instability requiring deformity correction or ligament reconstruction, 3). Burn stiffness, 4). Active infection around the elbow joint and/or sepsis-related stiffness, and 5). Bony ankylosis.

By searching our hospital record, a total of 157 patients were matched the inclusion criteria and were subsequently short-listed for further interview. We took some important information from the hospital record, such as contact details, patient’s demographic information, aetiology of stiffness, preoperative ROM and functional status, neurological status and any complications related to the procedure (Table 1). The short-listed candidates were contacted via phone calls or social media and requested to visit the hospital for the follow-up. Out of 157 short-listed patients, 141 patients (male =90; female = 51) with 143 elbows (posttraumatic, *n* = 75; non-traumatic, *n* = 68) responded our calls and agreed to participate in the study. The average age of the participants was 33 years (12–58) at the time of surgery. All the patients in the posttraumatic group had unilateral stiffness, whereas two patients with RA in the non-traumatic group had bilateral elbow involvement. The average follow-up period of the patients was 44 months (24–90).

**Table 1:** Demographic characteristics of the patients with posttraumatic and non-traumatic stiffness (mean ± SD or *n*)

Parameters	Posttraumatic group (*n* = 75)	Non-traumatic group (*n* = 68)	*P* value
Age (years)	32.1 ± 10.3 (12–55)	34.7 ± 9.4 (17–58)	0.12
Male/Female (*n*)	52/23	38/30	
Dominant/Non-dominant (*n*)	48/27	40/28	
Duration of Stiffness (months)	18.9 ± 21.3 (6–120)	25.0 ± 19.6 (6–120)	0.08
Follow-up (months)	45.3 ± 19.3 (24–90)	44.2 ± 14.4 (24–81)	0.71
Etiology of Stiffness	Distal humerus fracture (31)	Synovial chondromatosis (19)	
	Proximal ulna fracture (19)	Overuse syndrome (17)	
	Proximal radius fracture (10)	Osteoarthritis (15)	
	Humerus and ulna fractures (6)	Rheumatoid arthritis (10)	
	Radius and Ulna fractures (4)	Tuberculosis (4)	
	Dislocation (3)	Gout (3)	
	Heterotrophic ossification (2)		

*SD* Standard Deviation; *n* Number of cases;

### Preoperative preparation

We performed preoperative imaging such as X-Ray and/or computed tomography (CT) scans for every elbow to carefully evaluate the nature of stiffness and to ensure that there is no apparent bony ankylosis. The preoperative ROM was evaluated carefully and ensured the possible location for release. All the procedures were performed by a single fellowship-trained senior orthopaedic surgeon using the same technique.

### Surgical procedure

**Fig. 1:**
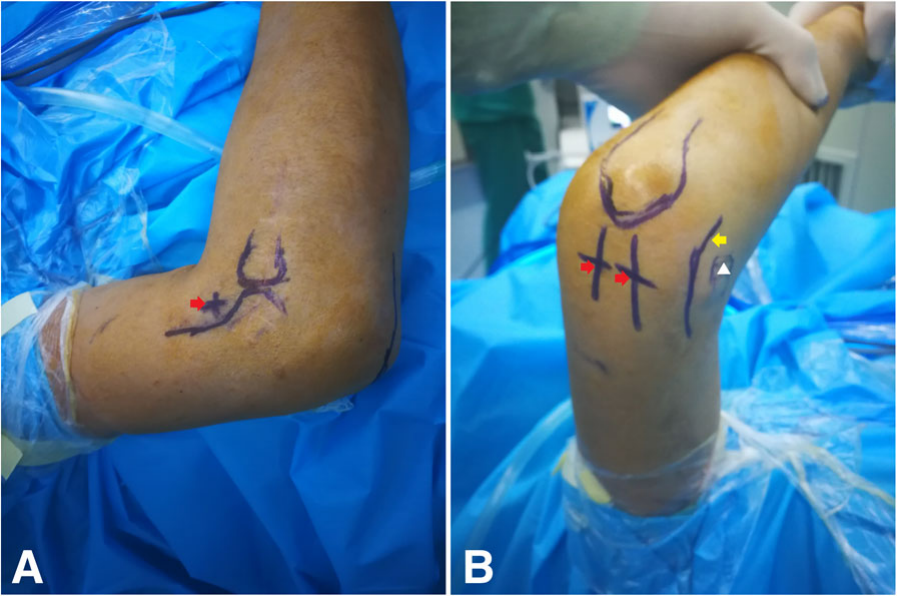
**a**-**b**. Anatomical landmarks. Bony landmarks from lateral view (**a**) show distal humerus, radial head and position of anterolateral portal (a red arrow head), and landmarks from posteromedial view (**b**) show olecranon process, positions of posteromedial and posterolateral portals (red arrowheads), medial epicondyle (a white triangle) and the course of ulnar nerve (a yellow arrow head)

The procedure was routinely performed under general anaesthesia in the supine position. The shoulder was abducted at 90° with a slight forward flexion to elevate the elbow slightly higher than the shoulder joint. The procedure involved marking all anatomical landmarks, such as the distal humerus, proximal radius, olecranon process, triceps muscle insertion, medial epicondyle, and most importantly the course of the ulnar nerve (Fig. 1). Before the intra-articular procedure, we performed ulnar neurolysis using a mini-open procedure in patients with prior ulnar nerve symptoms or patients needing removal of osteophytes and/or loose bodies around the ulnar groove.

Initially, 20 ml of saline was injected into the joint from the point just anterior to the radio-capitellar joint to achieve adequate joint distension. The point was well demarcated by an intersection of the anterior border of the distal humerus and anterior border of the proximal radius while the elbow was held at 90° flexion. In the case where elbow cannot be flexed at 90°, the demarcation was made at maximum flexion. Firstly, the anterolateral portal (ALP) was carefully created by making a 0.5 cm skin incision on the same point where the saline was injected, followed by insertion of an arthroscopic sheath with a trocar into the joint. The same device was, then, pushed further towards the medial subcutaneous tissue on the opposite side using the inside-out technique to create the anteromedial portal (AMP). Secondly, the radiofrequency (RF) device was inserted into the joint carefully with the guidance of arthroscopic sheath to prevent possible injury to the medial antebrachial nerve [19]. All fibrous scar tissues and hyperplastic synovium were debrided using RF device and a 4-mm shaver. Loose bodies were carefully extracted, and osteophytes around the coronoid fossa, radial fossa, and coronoid process were meticulously removed using a 3.5 mm arthroscopic burr. The large loose bodies and osteophytes were broken down into small pieces and taken out carefully. The anterior capsular contracture could be released if the stiffness was because of capsular contracture. However, there was always a risk of injuring radial nerve while performing anterior capsulectomy as it lies only about 1 to 2 mm from the anterior joint capsule. The risk was minimized by avoiding suction during capsulectomy especially around the radial head. Then, the posterior compartment was released using posterolateral approach. Two portals, posterolateral (PL) and posteromedial (PM), were created approximately 1 cm proximal to the olecranon process on either side of the triceps tendon. These portals allowed removing scars or osteophytes on posterior compartments especially in olecranon fossa and olecranon process in a similar way that the anterior compartment was released. If the contracture was because of the scar or osteophytes around the posteromedial aspect, great care was taken not to injure the ulnar nerve. This was better addressed by ulnar neurolysis. Sufficient release was identified by checking the ROM in all the planes, and acceptable elbow motion was achieved by joint manipulation.

Finally, a drainage tube was placed routinely, portals were closed, and the joint was stabilized with an orthotic device in extension [7].

### Rehabilitation

We used the same rehabilitation protocol for all the patients. Patients were instructed to do functional exercise on the first postoperative day under full dose of oral analgesics and buprenorphine skin patch analgesia. The functional exercise included elbow flexion and forearm rotation. The index elbow was placed on a continuous passive motion (CPM) machine three times a day each lasting for 30 min. The rate was progressively increased. Ice pack compression was used to minimize the swelling and used in between the exercise. Gradually, the patients were encouraged to perform active functional exercise followed by progressive muscle strengthening exercises. Patients were discharged with an orthotic device and oral Naproxen 200 mg three times a day for 2 months to prevent possible HO.

### Clinical evaluation

Patients were clinically assessed using both the subjective and objective evaluations according to Mayo Elbow Performance Index (MEPI) score [20], Visual Analogue Scale (VAS) and level of satisfaction. The MEPI assesses pain, ROM, stability, and function, whereas VAS assesses pain at rest. The level of satisfaction was measured by a self-constructed scale consisted of very satisfied, satisfied, neutral, dissatisfied and very dissatisfied.

**Fig. 2:**
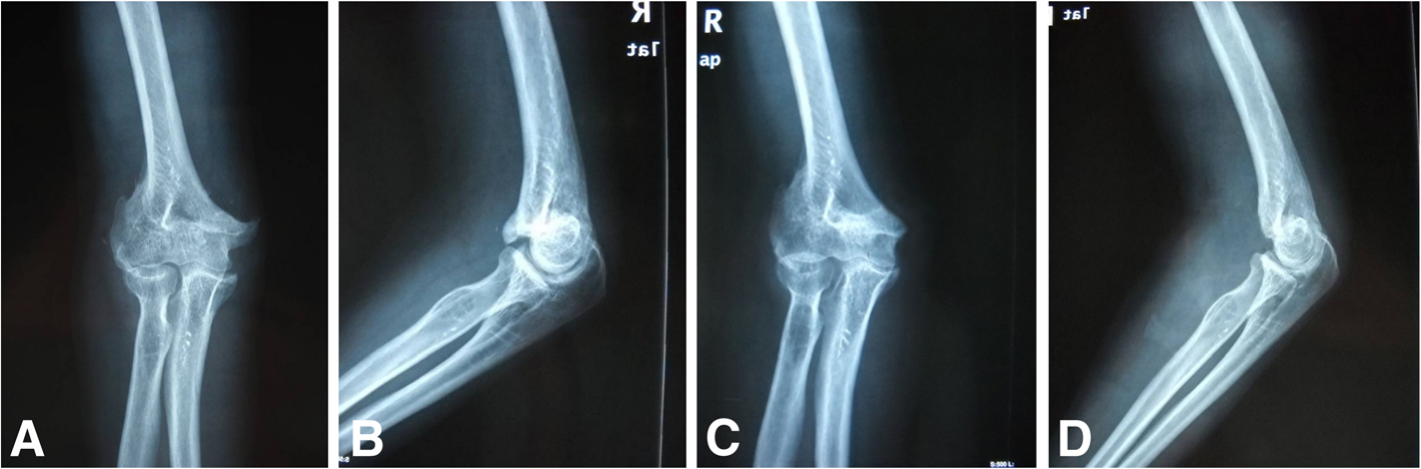
**a**-**d** Preoperative and postoperative radiographs of a 41-year-old male with 8 months old fracture. (**a**) and (**b**) are preoperative radiographs of anteroposterior (AP) and lateral view respectively; and (**c**) and (**d**) are postoperative radiographs. The ROM was improved significantly from 30° to 60° preoperatively to 10° to 110° postoperatively

Written informed consent to participate in the current study was taken from all the participants at the final follow-up. The subjective questionnaire was handed over to the patients and requested to fill up. The objective evaluations were performed by a single observer postoperatively. The elbow flexion and extension was carefully measured using a hand-held goniometer. Postoperative radiographs were also performed routinely to compare with preoperative radiographs (Fig. 2).

### Statistical analysis

We used IBM SPSS Statistics 23 for statistical analysis. *Chi-square* test or *Fisher’s exact* test was applied to analyze categorical variables, and *t*-test (two-tailed) was applied to analyze continuous variables. Nonparametric continuous variables were analyzed using the *Mann-Whitney U* test. Results of categorical variables were presented as frequencies and percentages whereas results of continuous variables were presented as the mean ± standard deviation (SD). A *P* value < 0.05 was considered a statistically significant difference. G Power 3.1.9.2 software was used to perform a post hoc power analysis of improved elbow ROM and MEPI as primary outcome measures.

## Results

### Clinical results

All the parameters including extension, flexion, ROM and MEPI scores were significantly improved postoperatively while compared to preoperative scores. In the posttraumatic group, the average extension was 38.6° ± 19.9° (0°-90°) preoperatively and 9.2° ± 8.4° (0°-30°) postoperatively (*P* < 0.01). The average flexion was 83.3° ± 21.8° (30°-120°) preoperatively and 117.9° ± 12.9° (75°-140°) postoperatively (*P* < 0.01). The average ROM was 44.6° ± 22.1° (0°-90°) preoperatively and 108.5° ± 18.5° (45°-140°) postoperatively (*P* < 0.01). The average MEPI score was 64.2 ± 11.7 (30–85) points preoperatively, and 91.0 ± 8.9 (55–100) points postoperatively (*P* < 0.01). According to MEPI score, 47 (62.7%) elbows were rated as excellent, 25 (33.3%) as good, 2 (2.7%) as fair and 1 (1.3%) as poor.

Similarly, in the non-traumatic group, the average extension was 35.6° ± 21.5° (10°-90°) preoperatively and 10.4 ± 16.4° (0–65) postoperatively (*P* < 0.01). The average flexion was 92.2° ± 22.5° (10°-115°) preoperatively and 121.9° ± 10.9° (90°-130°) postoperatively (*P* < 0.01). The average ROM was 56.3° ± 28.4° (0–100) preoperatively and 111.1° ± 21.5° (30°-140°) postoperatively (*P* < 0.01). The average MEPI score was 60.9 ± 14.4 (40–85) points preoperatively, and 87.6 ± 11.3 (60–100) points postoperatively (*P* < 0.01). According to MEPI score, 26 (38.3%) elbows were rated as excellent, 34 (50%) as good and 8 (11.8%) as fair. However, no statistically significant differences were observed in the rate of postoperative improvement of extension, flexion, ROM and MEPI score in both the groups (Table 2).

**Table 2:** Clinical and functional improvement of both groups at the last follow-up (mean ± SD)

Parameters	Posttraumatic group (*n* = 75)	Non-traumatic group (*n* = 68)	*P*-value
Extension	29.4 ± 20.6 (− 15–85)	25.2 ± 24.5 (− 30–90)	0.27
Flexion	34.6 ± 23.9 (0–100)	29.7 ± 26.2 (−20–115)	0.29
ROM	63.8 ± 26.3 (−5–115)	54.5 ± 35.7 (− 20–115)	0.08
MEPI	29.0 ± 11.5 (0–60)	26.6 ± 11.3 (5–55)	0.21

No statistical significant differences were observed in all the parameters

*SD* Standard Deviation; *n* Number of cases; *ROM* Range of motion; *MEPI* Mayo elbow performance index;

A post hoc power analysis of improved ROM and MEPI score showed the statistical power of 41 and 23%, respectively. It signified that a very large sample size would be required to detect statistically significant differences.

Thirteen cases with severe stiffness in the posttraumatic group and three cases in the non-traumatic group underwent ulnar neurolysis. Among them, 5 cases in the posttraumatic group and 1 case in the non-traumatic group had preoperative paraesthesia over ulnar nerve distribution. Remaining patients underwent neurolysis for scar, osteophytes or loose bodies removal around the posteromedial side including the ulnar groove. All of the patients recovered well without symptoms.

### Visual analogue scale (VAS)

The postoperative VAS score at rest was significantly improved (*P* < 0.05), and are well illustrated in Table 3.

**Table 3:** Preoperative and postoperative VAS pain at rest (n, %)

VAS Pain	Posttraumatic group (*n* = 75)		Non-traumatic group (*n* = 68)	
	Preoperative	Postoperative	Preoperative	Postoperative
0	16 (21)	55 (73)	6 (9)	25 (37)
1	42 (56)	20 (27)	18 (26)	37 (54)
2	17 (23)	0 (0)	30 (44)	6 (9)
3	0 (0)	0 (0)	14 (21)	0 (0)

*n* Number of cases; *VAS* Visual analogue scale

### Satisfaction

No statistically significant difference was observed in the level of satisfaction in both the groups (*P* = 0.76). In the posttraumatic group, 41 (54.7%) cases were very satisfied, 29 (38.7%) were satisfied, 2 (2.7%) were neutral, 2 (2.7%) were dissatisfied, and 1 (1.3%) was very dissatisfied. Similarly, in the non-traumatic group, 37 (54.5%) cases were very satisfied, 23 (33.8%) were satisfied, 2 (2.9%) were neutral, 5 (7.3%) were dissatisfied, and 1 (1.5%) was very dissatisfied.

### Complications

The overall complications of our cohorts were 9% (Table 4). In the posttraumatic group, 3 cases suffered from transient ulnar nerve palsy. However, all recovered spontaneously within 6 months. Similarly, 3 cases reported no improvement in the flexion-extension elbow motion and required for second intervention. The most common reason for re-stiffness was the failure to comply with the strict postoperative rehabilitation program (*n* = 2) followed by HO (*n* = 1). Two patients including 1 HO needed arthroscopic revision surgery and reported a satisfactory ROM. One patient was successfully treated with MUA. One superficial portal site infection occurred and was treated with oral antibiotics successfully. In the non-traumatic group, 1 woman suffered from transient ulnar nerve palsy postoperatively. This patient had significant elbow stiffness (extension 35° and flexion 80°) of her dominant elbow for 6.5 years preoperatively. She needed an extensive scar and osteophytes removal from the posteromedial elbow to achieve adequate flexion. However, she gained a significant elbow ROM (extension 10° and flexion 130°) improvement. Her symptom was recovered spontaneously in 3 months. Three cases did not improve their elbow ROM postoperatively. Two patients underwent arthroscopic revision surgery, and they improved satisfactorily. Regardless of minimal improvement of elbow motion postoperatively, one RA patient on non-dominant elbow refused for second surgery as she was satisfied with the previous arthroscopic arthrolysis in terms of pain relief. Two superficial wound infections occurred and were treated successfully with oral antibiotics.

**Table 4:** Comparison of complication rate between posttraumatic and non-traumatic stiffness (n, %)

Complications	Posttraumatic group (*n* = 75)	Non-traumatic group (*n* = 68)	*P* value
Superficial wound infection	1 (1.3)	2 (2.9)	0.9
Transient ulnar nerve palsy	3 (4.0)	1 (1.5)	
Recurrence			
Required reoperation	2 (2.7)	2 (3)	
Required MUA	1 (1.3)	0 (0.0)	
No intervention	0 (0.0)	1 (1.5)	

No statistically significant difference was observed in complications

*n* Number of cases;

No case of the permanent ulnar nerve, transient or permanent median or radial nerve palsy, instability, and deep infection was observed in both the groups.

## Discussion

The most important finding of our study was that arthroscopic arthrolysis of posttraumatic and non-traumatic elbow stiffness offers a significant and comparable improvement in terms of clinical and functional outcomes, and the level of satisfaction. The ulnar neurolysis was effective in prior ulnar symptoms and severe stiffness requiring an extensive release, especially in the posteromedial aspect.

We performed arthroscopic arthrolysis regardless of a relatively poor preoperative elbow ROM mainly because of aesthetic concern in the young individuals. Our patients achieved significant postoperative improvements of 64° in the posttraumatic group and 54° in the non-traumatic group. This finding was consistent or even better as compared with the previous literature of average elbow ROM improvement of 39° (24°to 66°) in the posttraumatic [3–5, 7, 8, 14, 21]; and 24°(16° to 36°) in the non-traumatic group [4, 5, 8]. Kim et al. [4] compared the similar etiological groups including 63 patients with posttraumatic (*n* = 33) and degenerative (*n* = 30) elbow stiffness, and followed-up for an average of 42.5 months with a minimum of 2 years. The average improvement of ROM was 50° in the posttraumatic group and 36° in the degenerative group. Although there was a statistically significant difference in preoperative ROM between the groups, they did not find any difference postoperatively. More recently, Willinger et al. [8] and Pederzini et al. [5] reported similar results of a significant improvement in ROM. Pederzini et al. [5] found a significant improvement in patients who have preoperative ROM of less than 80°.

Our result supports the fact that the postoperative ROM significantly improves after arthroscopic arthrolysis of elbow stiffness. However, the degree of improvement was greater in the posttraumatic group that’s perhaps because of the following reasons. First, preoperative elbow motion of posttraumatic stiffness is relatively severe and more room for improvement is available. Second, as Kim et al. [4] stated, the patients in posttraumatic stiffness groups are relatively active and younger having more aesthetic concerns. They are motivated enough to accomplish strict postoperative rehabilitation, which is essential for optimum results. Third, most of the patients with non-traumatic stiffness are generally the result of chronic inflammatory or degenerative joint diseases with a prolonged course, so the joints are usually weaker, osteopenic and full of osteophytes. Less postoperative elbow ROM in this group could be due to an incomplete osteophytes removal and lack of vigorous manipulation with fear of possible fracture of relatively weak bones intraoperatively.

Not only the elbow motion but also the improvement of elbow functions and reduction of pain are equally crucial after arthrolysis for ADL and return to pre-injury level. Both pain and function improved significantly after elbow arthrolysis. The MEPI scores improvement was comparable in both the groups and 96% of the patients in the posttraumatic group, and 89% of the patients in the non-traumatic group were rated as good to excellent postoperatively. However, MEPI score in the non-traumatic group was slightly lower than in the posttraumatic group. Our results were consistent with the result of Willinger et al. [8]. Most of their patients improved significantly with good to excellent results. In contrast, despite significant postoperative improvements from 60 to 81 points and 65 to 91 points in posttraumatic and degenerative stiffness respectively, Pederzini et al. [5] reported a significantly higher score in the degenerative group than in the posttraumatic group. It might be because of differences in the patient selections as we included not only the degenerative stiffness but also the inflammatory diseases such as RA. Despite having a slightly minimal improvement in elbow motion, most RA patients reported a marked reduction of pain following surgery. Most of them were satisfied with the procedure, and they would choose a similar procedure if required.

The elbow arthroscopy has many inherent complications. These complications are frequently related to the proximity of the arthroscopic portals to the neurovascular structures and limited working space [22–25]. The overall complication after elbow arthroscopy occurs up to 16% and half of them consists of neurological complications [7, 19]. The most common nerve that predisposes to injury is the ulnar nerve [5, 7, 21]. However, other nerves such as median nerve and radial nerve have also been reported to be affected equally [4, 5, 13]. Pederzini et al. [5] reported 2.2% of neurological complications, including 2 ulnar nerve and 3 (posterior interosseous nerve (PIN) palsies in the posttraumatic group. The ulnar nerve complications occurred in patients who did not undergo ulnar neurolysis. Later, these patients were treated successfully by neurolysis [5]. The reason for PIN injuries was because of traction or suction. Wu et al. [7] even reported up to 10% of transient ulnar nerve palsy. However, Kim et al. [4] reported 2 transient median nerve paraesthesia in patients whose large loose bodies were extracted with difficulty. In our study, the overall complication rate was 9%; and 2.7% of the complications comprised of transient ulnar nerve palsy. Those nerve palsies occurred in the early years of arthroscopic arthrolysis in patients with a significant preoperative stiffness. The reason we considered was the thermal injury by the radiofrequency device during the removal of scar around the medial aspect of the elbow or forceful extraction of osteophytes/loose bodies from the ulnar groove. So, we later started to perform ulnar neurolysis using a mini-open procedure in patients needing posteromedial aspect intervention.

Many patients with severe elbow stiffness suffer ulnar nerve palsy preoperatively. The reason might be direct nerve compression by scar tissue and osteophytes and/or presence of loose bodies in the ulnar groove. Some authors advocate for routine ulnar nerve decompression in patients with ROM less than 100° [21, 26]. In Pederzini et al.’s [5] report, the ulnar neurolysis was adequate for 94% of the symptomatic patients without requiring anterior transposition. Similarly, Willinger et al. [8] also performed ulnar neurolysis in 12 (28.6%) patients who have severe elbow stiffness. All recovered well without any ulnar nerve symptoms. We also found a similar result in our cohort that all the patients who received ulnar neurolysis improved satisfactorily without postoperative symptoms.

Failure to comply with strict postoperative rehabilitation protocol or incomplete scar and/or osteophytes removal is the primary cause of failure to attain adequate elbow motion [4, 7]. The postoperative rehabilitation is essential to reduce pain and swelling and to strengthen arm muscles. Despite an acceptable intraoperative elbow ROM, 3 patients in each group did not improve postoperatively. We considered the cause to be inadequate postoperative rehabilitation. Therefore, it should be started without delay to maintain intraoperative elbow motion, attain optimal outcome and minimize the recurrence [4, 7, 9].

The strength of this study is a comparative study of arthroscopic arthrolysis between the posttraumatic and non-traumatic aetiology in a relatively large sample size with a significant stiffness. However, several limitations exist. First, this study embraces all biases related to a retrospective study. Second, there is the possibility of institutional and regional bias as this is the report from a single hospital. Last but not least, despite prescribing similar rehabilitation protocol, some patients could have adopted different quality and consistency of rehabilitation which perhaps affected the clinical outcome of the surgery.

## Conclusion

This study demonstrates that despite having a severe stiffness, mid-term follow-up of arthroscopic arthrolysis in posttraumatic and non-traumatic elbow stiffness offers satisfactory clinical outcomes. There were significant and comparable clinical improvements in both groups; however, the rate of improvement was higher in the posttraumatic group. Most of the patients were satisfied with the surgical procedure. Considering this report, the authors conclude that arthroscopic arthrolysis is an effective minimally invasive procedure and a good option for the treatment of elbow stiffness, but it is not without complications. The postoperative rehabilitation is equally essential to maintain intraoperative elbow ROM, to attain optimal outcome and to prevent complications.

## Data Availability

The datasets supporting the conclusion of this article are included within the article. Upon request, raw data can be provided by the corresponding author.

## Abbreviations

ADL: Activity of daily living
ALP: Anterolateral portal
AMP: Anteromedial portal
CMP: Continuous passive motion
CT: Computed tomography
HO: Heterotrophic ossification
MEPI: Mayo Elbow Performance Index
QoL: Quality of life
RA: Rheumatoid Arthritis
RF: Radiofrequency
ROM: Range of motion
SD: Standard deviation
VAS: Visual analogue scale
